# Supplementary material for: Mechanisms governing the pioneering and redistribution capabilities of the non-classical pioneer PU.1
Source: Nat Commun. 2020 Jan 21;11:402. doi: 10.1038/s41467-019-13960-2 (PMC6972792; doi:10.1038/s41467-019-13960-2)
Supplement: Supplementary file 7 — Source data [file 41467_2019_13960_MOESM7_ESM.zip › Source_Data/Figure6/Figure6B_MotifScanOutput/FLI1_mutPU.1/homerResults/motif23.similar.html]

motif23

## Information for motif23

A
C
G
T
A
C
G
T
A
C
G
T
A
C
T
G
C
G
T
A
C
G
T
A
A
T
C
G
  
Reverse Opposite:  

A
T
G
C
C
G
A
T
A
C
G
T
A
G
T
C
C
G
T
A
C
G
T
A
C
G
T
A
  

|  |  |
| --- | --- |
| p-value: | 1e-45 |
| log p-value: | -1.045e+02 |
| Information Content per bp: | 1.979 |
| Number of Target Sequences with motif | 936.0 |
| Percentage of Target Sequences with motif | 9.12% |
| Number of Background Sequences with motif | 1973.4 |
| Percentage of Background Sequences with motif | 5.18% |
| Average Position of motif in Targets | 100.7 +/- 53.8bp |
| Average Position of motif in Background | 98.7 +/- 63.4bp |
| Strand Bias (log2 ratio + to - strand density) | 0.1 |
| Multiplicity (# of sites on avg that occur together) | 1.07 |
| Motif File: | file (matrix) reverse opposite |

### Similar de novo motifs found

|  |  |  |  |  |  |  |  |
| --- | --- | --- | --- | --- | --- | --- | --- |
| Rank | Match Score | Redundant Motif | P-value | log P-value | % of Targets | % of Background | Motif file |
| 1 | 0.827 | G A T C C G A T A G C T A G C T T A C G G T C A G C T A C T A G T G A C G A C T A T G C | 1e-23 | -54.842453 | 2.94% | 1.38% | motif file (matrix) |
| 2 | 0.766 | T A G C C G A T C G A T A G C T A C T G C G T A G C T A A T C G A T G C C G A T A T G C A C G T | 1e-15 | -36.022358 | 0.33% | 0.02% | motif file (matrix) |
